# Enhanced Biofuel Cells Based on a Hybrid Enzymatic/Bimetallic Composite for Complete Lactate Catalytic Electrooxidation

**DOI:** 10.1021/acsmaterialsau.5c00039

**Published:** 2025-06-09

**Authors:** Jefferson Honorio Franco, João Victor Bonaldo, Shelley D. Minteer, Adalgisa R. De Andrade

**Affiliations:** † Department of Chemistry, Faculty of Philosophy Sciences and Letters at Ribeirão Preto, 124588 University of São Paulo, Ribeirão Preto, São Paulo 14040-901, Brazil; ‡ National Institute for Alternative Technologies of Detection, Toxicological Evaluation and Removal of Micropollutants and Radioactives (INCT-DATREM), Institute of Chemistry, UNESP, P.O. Box 355, Araraquara, São Paulo 14800-900, Brazil; § Department of Chemistry, 14717 University of Utah, Salt Lake, Utah 84112, United States; ∥ Department of Chemistry, Missouri University of Science and Technology, Rolla, Missouri 65401, United States

**Keywords:** enzymatic biofuel cell, energy generation, metallic catalyst, oxalate oxidase, lactate oxidation

## Abstract

We describe complete lactate electrooxidation in an enzymatic biofuel cell that combines the catalytic action of the bimetallic composite Ru@Pt-CNT and the enzyme oxalate oxidase (OxOx). The Ru@Pt-CNT/OxOx hybrid electrode was 2.0-fold more catalytically active than the electrode containing the bimetallic composite only. During chronoamperometric experiments, the hybrid electrode achieved a 35% higher maximum current density (2.65 ± 0.15 mA cm^–2^) than the Ru@Pt-CNT electrode. Electrochemical impedance spectroscopy showed that the hybrid electrode had lower charge transfer resistance than the Ru@Pt-CNT electrode, confirming that OxOx had a high affinity for lactate during the bioelectrocatalytic reaction on the electrode surface. Furthermore, 18-h long-term bulk electrolysis revealed that lactate electrooxidation at the Ru@Pt-CNT/OxOx hybrid electrode provided a total charge of 1.2 ± 0.2 C, which was 3-fold higher than the total charge generated by the Ru@Pt-CNT electrode. The lactate oxidation products generated at the hybrid electrode were detected during bulk electrolysis by chromatography, which showed that the hybrid biofilm harvested all 10 electrons from lactate, completely oxidizing it to CO_2_. With exceptional stability and catalytic performance, the hybrid electrode acted in the multiple catabolic steps of lactate oxidation. Overall, the interaction between Ru@Pt-CNT and OxOx enhanced the assembly of lactate biofuel cells to improve lactate electrooxidation. This could pave the way for developing efficient electronic devices with promising applications in bioelectrochemistry.

## Graphical abstract

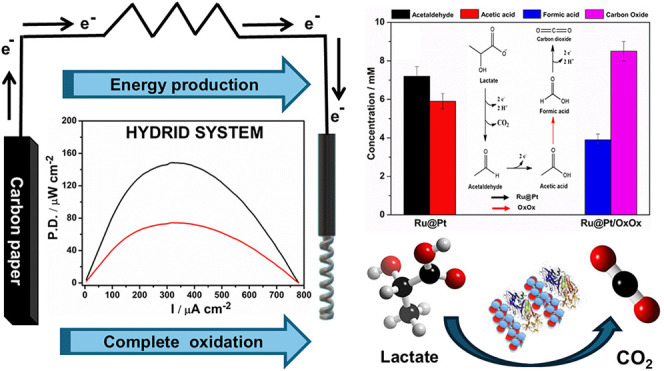

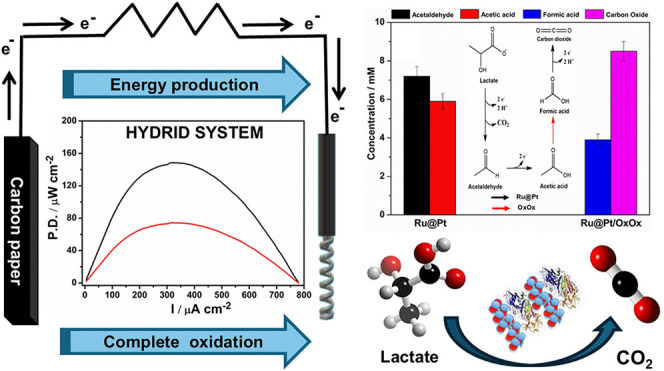

## 1 Introduction

Enzymatic biofuel cells (EBFCs) are promising for collecting electrons and generating electrical energy from fuels.^1^ Lactate is one of the most attractive metabolites for fuel in EBFCs:^2^ high solubility and excellent theoretical energy density (3.041 W h L^–1^) make it an ideal candidate for generating energy in EBFCs.^3^ Also, high lactate concentration and accessibility in human sweat have allowed stable epidermal EBFCs to be developed and worn as healthcare devices on the skin.^4^ Lactate-based biofuel cells (LBFCs) can potentially be employed to develop self-powered lactate biosensors,^5^ drug delivery systems,^6^ wearable bioelectronics,^7^ and bioenergy harvesters for powering electronics.^8,9^ Most literature studies have used just one enzyme, mainly lactate oxidase (LOx), to catalyze lactate electrooxidation and generate energy from LBFCs.^10^ However, LOx only catalyzes the first catabolic step of lactate oxidation.^8^ Adding more enzymes to LBFCs could enhance the current and power density, improve the catalytic activity by increasing the electron transfer rate, and allow the collection of more electrons from lactate.^11,12^ However, increasing the number of enzymes on the electrode surface may create an unstable film, decreasing the lifetime of the EBFCs.^13^ Hybrid systems can overcome this drawback. At the same time, the enzyme cleaves the fuel’s C–C bonds, and the organic catalysts^14^ or metallic nanocatalysts^15,16^ catalyze the oxidation of oxygen, nitrogen, or sulfur-containing functional groups and their intermediates.^17^ These represent a simple and efficient way to enhance EBFC performance by improving the electrode stability, lifetime, and fuel electrooxidation rate.^14,18,19^ This also overcomes the great difficulty in obtaining the breaking of the C–C bond in metallic electrocatalysts.^20^

Oxalate oxidase (OxOx) is well-known to cleave carbon–carbon (C–C) bonds of simple carboxylic acids;^21,22^ however, OxOx cannot be used to catalyze lactate directly,^15^ it needs an initial catalyst to optimize the oxidative steps.^17,23^ When combined with an initial catalyst, OxOx has demonstrated exceptional efficiency in oxidizing various intermediates during electrolysis.^15,16,24,25^

Materials science has improved the performance of metallic nanomaterial electrodes in electrocatalysis.^16,26^ For example, carbon nanotube (CNT) nanocomposites based on noble metals, including Pt/CNT, Ru–Pt/CNT, and Sn–Pt/CNT, have been used to modify electrodes, generating high catalytic activity in microbial fuel cells (MFCs).^27^ The modified electrode increased MFC performance 9-fold compared to the graphite electrode-based MFC. Nanofluids can also channel bacterial energy more efficiently and generate more power.^27,28^ Table S1 highlights hybrid systems with good power density, current density, and complete fuel electrooxidation for lactate,^12^ glucose,^15^ ethylene glycol,^16,24^ ethanol,^29^ −,^30,31^ and glycerol.^14,25^

Ru–Pt electrocatalysts have attracted attention because they are active anodic electrocatalysts in direct methanol fuel cells (DMFCs)^32^ glycerol,^33^ and ethanol.^34^

According to Da Silva et al.,^35^ incorporating ruthenium into platinum shifts the oxidation potential toward more negative values than pure platinum, highlighting the benefits of incorporating a second metal into the electrocatalyst composition. This characteristic is attributed to an increased density of active catalytic sites on the electrocatalyst surface, facilitating enhanced substrate molecule oxidation.^35^ Consequently, the Ru@Pt/CNTs bimetallic system exhibits enhanced thermodynamic and catalytic performance,^36,37^ making it a promising candidate for biofuel cell applications.

Despite several studies on the adequate preparation of Pt–Ru electrocatalysts supported by carbon nanotubes,^36,37^ a comprehensive investigation of this bimetallic catalyst in biofuel cells has not been reported in the literature.

Furthermore, fabricating core–shell nanocatalysts is an economically effective and reliable method to obtain an active metal with a large surface area and tunable and ordered properties.^38^ Developing electrocatalysts bearing a bilayer consisting of Ru@Pt nanoparticles increases tolerance to carbon monoxide (CO) and corrosion resistance, solving the dilemma of using dissolution-prone metals to alleviate the CO poisoning effect.^39^ This approach provides a well-ordered core–shell structure, paving the way for commercializing low-temperature fuel cells that employ inexpensive reformates (H_2_ with CO impurity) as fuel.^39^

Herein, we have developed an enhanced bicatalytic hybrid electrode containing the bimetallic composite catalyst Ru@Pt-CNT and the enzyme oxalate oxidase (OxOx) to achieve complete lactate electrooxidation. We performed electrochemical experiments to evaluate the catalytic activity and ability of the hybrid electrode to generate energy. The lactate oxidation products obtained after long-term electrolysis were detected and identified by high-performance liquid chromatography (HPLC).

## 2 Experimental Section

### 2.1 Chemicals

Lactate, acetaldehyde, acetic acid, formic acid, and sodium carbonate were purchased from Sigma-Aldrich. Citric acid-phosphate buffer (150 mmol L^–1^, pH = 5.2) was prepared by dissolving the salts in ultrapure water. All the reagents were of analytical grade and used without further purification. All the solutions were prepared using high-purity water from the Millipore Milli-Q system.

### 2.2 Oxalate Oxidase (OxOx) Expression and Purification

As previously reported, the pPICZαA vector expressed the OxOx enzyme gene.^40^ Cells transformed into KM71H by electroporation were selected based on resistance to Zeocin.^30,40^ As reported before, to increase OxOx production, was employed to reproduce the plasmid responsible for producing the enzyme on a larger scale.^15^ Thus, eight colonies containing were chosen and cultivated in an MMB induction culture medium (100 mM potassium phosphate buffer, pH 6, yeast nitrogen base with 1.34% ammonium sulfate, 4 × 10^–5^ g·L^–1^ biotin); 0.5% methanol. This procedure was performed every 24 h for 3 days. As a negative control for the enzymatic assay, a KM71H strain was transformed with an empty pPICZαA vector and cultured under the above-mentioned conditions.

Finally, the centrifugation was performed at 8,000*g* for 5 min, and the final supernatant (OxOx extract) was separated into the enzymatic assays. Then, the solution containing the enzyme was concentrated by an ultrafiltration process (Labscale TFF), employing a 50 cm^2^ Biomax membrane to retain proteins larger than 5 kDa, followed by purification by SDS-PAGE (sodium dodecyl sulfate-polyacrylamide gel electrophoresis).^15^ Subsequently, the OxOx was stored in individual 50-μL Eppendorf tubes at −80 °C for future use.

### 2.3 Enzymatic Activity Assay

Enzymatic activity was evaluated using the method proposed by Requena and Bornemann.^41^ The Bradford method determined the protein concentration;^42^ a bovine serum albumin standard curve was employed. The protein was quantified by reacting it with the Coomassie Blue 250 reagent, which gave a protein complex monitored at 650 nm. The final enzymatic extract was stored at −80 °C in separate 500-μL Eppendorf tubes until it was used; its specific activity was 499 ± 9 U/mg (μmol of substrate converted per minute per milligram of enzyme).^15^ All the enzyme solutions were freshly prepared and immediately used.

### 2.4 Ru@Pt-CNT Bimetallic Nanocatalysts Synthesis

The bimetallic composite Ru@Pt-CNT was synthesized as reported by Da Silva et al.^35^ To fabricate the desired core–shell nanocatalyst morphology, anionic surfactant sodium citrate dihydrate (SDS) was employed as a template for self-assembling positively charged Ru species into the core and negatively charged platinum species into the outer shell. Sodium citrate was used as a stabilizing agent to prevent nanoparticle aggregation. Ru@Pt catalyst synthesis aimed to obtain good homogeneity, stability, and complete reduction of the metals. The nanocatalysts were prepared with a constant metal-to-carbon ratio of 40:60 wt %, maintaining a 1:1 molar ratio between the different metal atoms in each case. The synthesis was conducted under a nitrogen atmosphere. A mixture of 40 mL ethanol, 30 mL 0.25 mol L^–1^ SDS, and 10 mL 20 mmol L^–1^ sodium citrate were stirred for 1 h to form and stabilize SDS micelles. Positively charged metal precursors were then added and stirred for another hour, followed by the addition of negatively charged platinum species. A freshly prepared cold sodium borohydride solution was rapidly added to reduce the self-organized metal precursors and form Ru@Pt nanoparticles. After 60 min, multiwalled carbon nanotubes (MWCNTs) were added and stirred for 12 h. To enhance binding sites and surface anchoring groups on the MWCNTs, they were functionalized by refluxing in a mixture of H_2_SO_4_/HNO_3_ acids, introducing hydrophilic hydroxyl and carboxyl groups. The electrocatalysts were filtered, washed with water and ethanol, and dried at 70 °C for 10 h.

### 2.5 Hybrid Electrocatalytic Electrode Preparation

The catalytic Ru@Pt-CNT, Nafion, and isopropanol ink followed the method described by Franco et al.^15^ and was prepared by sonicating, during 30 min, a mixture of 2 mg of Ru@Pt-CNT, 80 μL of isopropanol, and 20 μL of 10% Nafion (18.2 mg mL^–1^). The Ru@Pt-CNT/OxOx hybrid electrode was fabricated by incorporating 30 U mL^–1^ (55.4 mg mL^–1^) of OxOx into the final Ru@Pt-CNT solution and homogenized the mixture for 30 s. Before starting the experiments, all the electrodes were dried in a desiccator overnight.

For the cyclic voltammetry (CV) assays, 5 μL of the suspension was pipetted into a mechanically polished glassy carbon (GC, 7 mm diameter, area of 0.071 cm^2^). The chronoamperometry (CA), electrolysis, electrochemical impedance spectroscopy (EIS), and power density measurements were performed by adding 50 μL of the suspension onto the carbon paper support (Fuel Cell Earth; area of 1 cm^2^). The final concentration of OxOx on the surface area of glassy carbon and carbon paper was 1.5 U cm^–2^ and 0.011 U cm^–2^, respectively.

### 2.6 Electrochemical Experiments

All the electrochemical experiments were carried out using an AUTOLAB potentiostat/galvanostat (software NOVA 1.11) and a homemade three-electrode cell. The working electrode was Ru@Pt-CNT or Ru@Pt-CNT/OxOx modified on GC for the CV assays and Ru@Pt-CNT or Ru@Pt-CNT/OxOx modified on carbon paper for the CA, EIS, and power density measurements. The reference electrode was Ag/AgCl, Cl^–^ (sat); the counter electrode was platinum wire.

The CV assays were performed at a scan rate of 10 mV s^–1^ and step potential of 0.001 V in a potential window ranging from 0.00 to 1.0 V (vs Ag/AgCl) at 25 °C. CA titration curves were obtained at a fixed potential of 0.85 V vs Ag/AgCl with successive addition of lactate to the solution (the lactate concentration varied from 0 to 110 mmol L^–1^).

The 18-h electrolysis was accomplished at 0.85 V vs Ag/AgCl with 100 mmol L^–1^ lactate. A Nafion membrane separated the anode compartment pressed to a gaseous diffusion cathode containing 20% platinum (A6ELAT/BASF). The cathodic compartment was maintained in direct contact with air.

The EIS measurements were performed using an Autolab 302N instrument with a Frequency Response Analyzer (FRA) module; the spectrum was obtained at frequencies ranging from 10 kHz to 1 mHz. To help elucidate the mechanism and to evaluate how the enzyme OxOx influenced the electrode, the experiments were carried out at open circuit potential (OCP, 0.35 V vs Ag/AgCl) and the operational potential (0.85 V vs Ag/AgCl) with the addition of 100 mmol L^–1^ lactate. Data were simulated by applying the equivalent circuit method and the software Nova 2.1.4. All the models presented χ^2^ < 0.001.

The power density and OPC measurements were performed in a two-chamber electrochemical cell separated by a Nafion membrane.^43^ The homemade air-breathing cathode compartment comprised a gaseous diffusion membrane containing 20% platinum on Vulcan XC-72 hot-pressed into a proton exchange membrane (Nafion 212) (130 °C and 12.5 kgf cm^–2^ for 120 s). Therefore, the cathode was maintained in direct contact with air, and the anode compartment was filled with 10 mL of 150 mmol L^–1^ citrate-phosphate buffer (pH = 5.2) containing Ru@Pt-CNT or Ru@Pt-CNT/OxOx in the presence of 100 mmol L^–1^ lactate. Furthermore, the potential was monitored for 1 h until it stabilized, and the power density curves were calculated from the data recorded at 1 mV s^–^.^1^ All the electrochemical experiments were performed in triplicate.

### 2.7 Lactate Oxidation Product Identification after Bulk Electrolysis by Analytical Technique

High-performance liquid chromatography (HPLC) experiments were performed to identify the lactate oxidation products generated after bulk electrolysis for 18 h. The HPLC instrument comprised a Shimadzu model LC-10AT chromatograph containing two pumps and an automatic injector coupled to a double online detection system, namely ultraviolet (λ = 210 nm) and refractive index (RID = 10A) detectors. The optimized mobile phase consisted of 3.33 mmol L^–1^ sulfuric acid (H_2_SO_4_) at a constant flow rate of 0.6 mL min^–1^ with an injection volume of 20 μL. The Bio-Rad Chromatography Column (Aminex HPX-87H, 300 × 7.8 mm, 9 μm, 8% cross-linkage, pH range 5–9) was employed and operated at 30 °C.

The lactate oxidation products were identified and confirmed by comparison with commercial standards. We compare the retention times of the standard samples’ chromatographic peaks and oxidation products. The lactate oxidation products were quantified by applying the chromatographic peak area value in the linear equation obtained from the standard sample calibration curve.

The 0.1 mol L^–1^ NaOH concentration reveals CO_2_ formation, confirming complete lactate oxidation after bulk electrolysis. The RID detector subsequently detected this produced sodium carbonate (Na_2_CO_3_) after 5 min of reaction. The Na_2_CO_3_ chromatographic peak, resulting from the NaOH and CO_2_ reaction, was confirmed by comparison with a standard solution. All the analyses were carried out in triplicate.

## 3 Results and Discussion

To elucidate the composition and physicochemical properties of the Ru@Pt-CNT bimetallic nanocatalyst, we will review prior literature findings. Da Silva et al.^35^ reported that thermogravimetric analysis of the synthesized Ru@Pt-CNT particle revealed an atomic ratio of Ru_45_:Pt_55_, a value aligned with the desired nominal composition.

The thermogravimetric curves showed that the bimetallic nanocatalyst, Ru@Pt, indicated the presence of residual surfactant from the synthesis process, leading to a unique mass loss behavior between 250 and 350 °C.^35^ Additionally, the metallic catalyst exhibited a well-defined peak for the carbon substrate and platinum, which agreed with standard crystallographic planes. The average crystallite size obtained by the Scherrer equation for the Ru@Pt-CNT metal was 2.6 nm. The Ru@Pt-CNT nanocatalyst TEM analysis confirmed the formation of small clusters with approximately 20 nm diameter distributed heterogeneously on the MWCNT substrate. The generated nanoparticles exhibited a satisfactory spherical shape and size, achieving good microscopic results. Given these promising findings, the Ru@Pt-CNT metallic nanoparticle was chosen for subsequent experiments.

### 3.1 Electrochemical Measurements for Lactate Oxidation Catalyzed by the Ru@Pt-CNT or Ru@Pt-CNT/OxOx Electrode

Thus, the electrochemical characterization was performed in the absence and the presence of 100 mmol L^–1^ lactate to evaluate how the Ru@Pt-CNT and Ru@Pt-CNT/OxOx electrodes (Figure 1A–C). Figure 1A depicts the cyclic voltammograms obtained for each electrode in a 150 mmol L^–1^ citric acid-phosphate buffer (pH = 5.2) solution containing 100 mmol L^–1^ lactate at 10 mV s^–1^ from 0.0 to 1.0 V vs Ag/AgCl.

**Figure 1:**
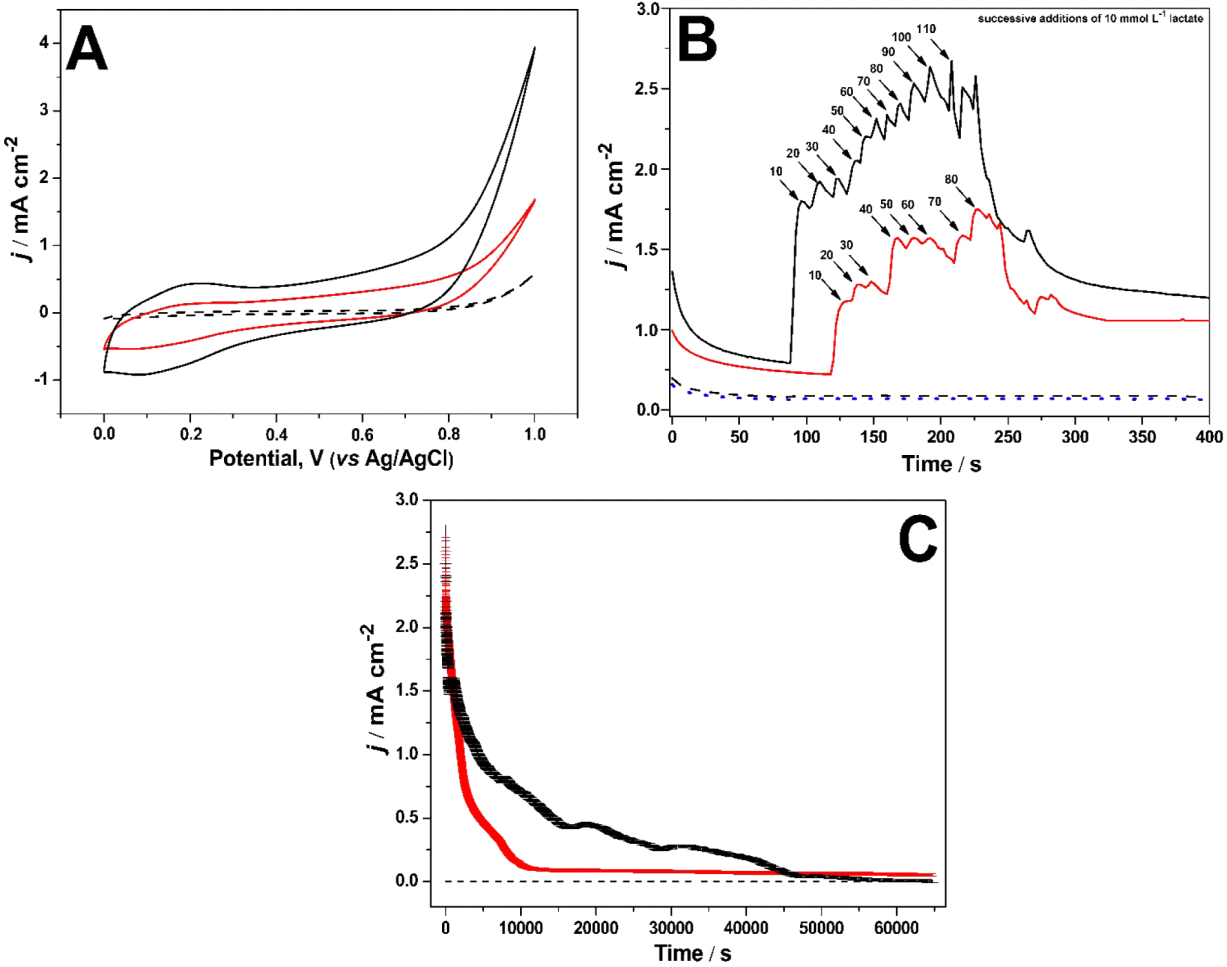
Electrochemical assays for the Ru@Pt-CNT (red line) and Ru@Pt-CNT/OxOx (black line) electrodes in the presence of 100 mmol L^–1^ lactate for cyclic voltammetry (A), chronoamperometric curve at 0.85 V vs Ag/AgCl (B), and long-term electrolysis at 0.85 V vs Ag/AgCl (C). The black dashed line in all the figures represents the hybrid electrode in the electrolyte in the absence of the fuel (lactate). The blue dotted line in Figure B represents the OxOx in the presence of lactate. Support electrolyte = 150 mmol L^–1^ citric acid-phosphate buffer (pH = 5.2), scan rate = 10 mV s^–1^, step potential = 0.001 V, and temperature = 25 °C.

To confirm that the Ru@Pt-CNT/OxOx hybrid electrode can catalyze lactate electrooxidation and to obtain essential information about the action of each catalyst employed in the LBFC, we accomplished CV assays for different electrode configurations (Figure 1A). The hybrid electrode (Ru@Pt-CNT/OxOx) exhibited a current density of 1.5 ± 0.1 mA cm^–2^ at an onset potential of 0.85 V, which is 2.3-fold higher than the 0.65 ± 0.02 mA cm^–2^ observed for the electrode containing only the bimetallic catalyst (Ru@Pt-CNT).

The current density increased for the Ru@Pt-CNT (red line) and Ru@Pt-CNT/OxOx (black line) electrodes upon successive 10 mmol L^–1^ lactate additions (Figure 1B). The hybrid electrode was more catalytically active than the Ru@Pt-CNT electrode, generating a maximum current density of 2.65 ± 0.15 mA cm^–2^ after 11 successive additions of 10 mmol L^–1^ lactate (lactate concentrations ranged from 0 to 110 mmol L^–1^). The Ru@Pt-CNT electrode provided a maximum current density of 1.75 ± 0.12 mA cm^–2^ after eight successive additions of 10 mmol L^–1^ lactate. Both electrodes presented linear amperometric responses upon rising lactate concentration until they reached a steady state. Moreover, the hybrid electrode had a 35% higher current density than the Ru@Pt-CNT electrode.

Moreover, Figure 1B shows that OxOx alone is not active in cleaving the C–C bond of lactate (blue dotted line). This also confirms the literature report that the OxOx enzyme is also inactive for ethylene glycol,^16,17^ ethanol,^30^ glucose,^15^ and lactate.^12^ Franco et al.^30^ reported that the bioanode containing only OxOx exclusively oxidized acetic acid, with an onset oxidation potential of 0.60 V vs SCE.

Therefore, the electrocatalytic profile observed in Figure 1B confirms the cooperative effect between OxOx and the bimetallic composite. OxOx cleaves the C–C bond of the products formed by enhancing catalytic activity and facilitating more electron collection from lactate molecules. This aligns well with published data on the combined effect of catalysts and enzymes in enhancing EFBC catalytic activity through deeper lactate oxidation.^12,15^

Next, we performed 18-h bulk electrolysis to determine the stability of the Ru@Pt-CNT and Ru@Pt-CNT/OxOx electrodes and the total charge and current density generated during lactate electrooxidation. Figure 1C shows the bulk electrolysis carried out for 18 h at a fixed potential of 0.85 V (vs Ag/AgCl) for the Ru@Pt-CNT (red line) and Ru@Pt-CNT/OxOx (black line) electrodes in the presence of 100 mmol L^–1^ lactate. Based on the amperometric profile in Figure 1C, the hybrid electrode was more active than the electrode containing the bimetallic composite. Thus, the hybrid electrode furnished a higher current density than the Ru@Pt-CNT electrode through improved lactate electrooxidation rates. Indeed, the hybrid electrode was stable and generated a total charge of 1.2 ± 0.2 C, 3-fold the total charge generated when the electrode containing the bimetallic composite was used only. Thus, the considerably higher catalytic activity of LBFC revealed the exceptional performance of the hybrid electrode.

The hybrid system consistently outperformed the system containing solely the bimetallic catalyst in energy production across all employed electrochemical techniques. This enhanced performance can be attributed to the cooperative action between the OxOx enzyme and the RuPt-CNT catalyst, facilitated by their optimal ratio of 3:1. This ratio corresponds to a 3-fold higher concentration of the enzyme relative to the organic catalyst. Additionally, recent studies have reported that OxOx enhances fuel oxidation after long-term electrolysis.^17,24^

Franco et al.^15^ reported the complete oxidation of glucose using a Ni@Pt-CNT hybrid system, the nanometallic catalyst exhibits superior efficiency in oxidizing oxygen groups compared to the enzyme’s substrate oxidation capabilities. Conversely, the enzyme acts specifically on a particular substrate and exhibits slower reaction rates in catabolic processes.^15^ A higher enzyme concentration is necessary to establish a balanced catalytic role for both components in lactate electrooxidation. The decreased catalytic current observed during 18-h electrolysis with a lower enzyme concentration can be attributed to this imbalance. Recent studies involving decarboxylase enzymes highlight the potential detrimental effects of excessively high enzyme concentrations, leading to system damage and reduced electrocatalytic performance. Consequently, a standardized OxOx enzyme concentration is consistently employed in BFC hybrid systems to maintain optimal performance.^15,16^ In conclusion, the superior performance of the hybrid system was evident when compared to previous studies on lactate oxidation.^44,45^

### 3.2 Identification and Quantification of Lactate Oxidation Products by HPLC-UV/RID

To understand the role that each catalyst (enzyme and bimetallic composite) plays in lactate electrooxidation and to propose a catalytic route, we carried out a chromatographic analysis to identify and quantify the lactate oxidation products generated after long-term electrolysis at the Ru@Pt-CNT or Ru@Pt-CNT/OxOx electrode. Lactate consumption during electrolysis was also evaluated for both electrodes assembled. Figure S1 illustrates the lactate consumption after prolonged electrolysis. The hybrid system exhibited superior performance, consuming 65% lactate compared to the bimetallic system, reaching a maximum consumption of 40%.

Figure 2A shows the HPLC profile of the lactate oxidation products obtained in the case of the Ru@Pt-CNT or Ru@Pt-CNT/OxOx electrode before (time = 0) and after electrolysis for 18 h. Product identification and quantification were confirmed by comparison with commercial standards. First, we analyzed the hybrid system in the supporting electrolyte (SE) (Figure 2Aa). No oxidation product peaks were detected at the end of the electrolysis, indicating that the SE is inert at the bimetallic composite and OxOx electrode. We also tested the lactate electrolysis at an electrode containing only the OxOx enzyme (Figure 2Ab). Under this condition, no oxidation products were detected, confirming that the Ox-Ox enzyme cannot be used directly to catalyze lactate oxidation. After long-term electrolysis, the Ru@Pt-CNT electrode generated two lactate oxidation products: acetaldehyde (7.2 ± 0.5 mmol L^–1^, two-electron oxidation) and acetic acid (5.9 ± 0.4 mmol L^–1^, four-electron oxidation), referring to peaks 2 and 3, respectively (Figure 2Ad). The hybrid electrode also afforded two more oxidable lactate products after electrolysis for 18 h: formic acid (3.9 ± 0.3 mmol L^–1^, four-electron oxidation) and carbon dioxide (8.5 ± 0.5 mmol L^–1^, 12-electron oxidation), referring to peaks 4 and 5, respectively (Figure 2Af).

**Figure 2:**
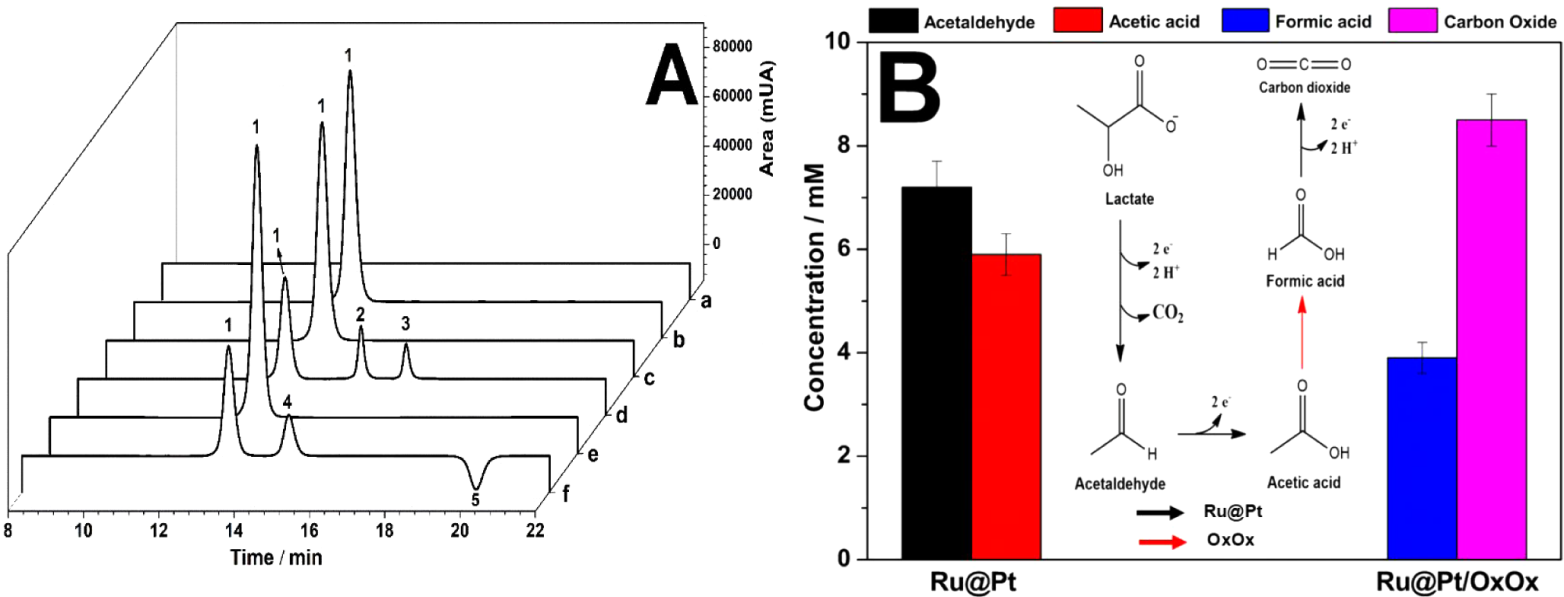
(A) Chromatograms of the lactate and the electrooxidation products: (a) supporting electrolyte with hybrid electrode (*t* = 18h), (b) electrode containing only OxOx with 100 mmol L^–1^ lactate (*t* = 18h), Ru@Pt-CNT electrode: (c) before (*t* = 0h), and (d) after (*t* = 18 h) bulk electrolysis with mmol L^–1^ lactate. Hybrid Ru@Pt-CNT/OxOx electrode: (e) before and (f) after bulk electrolysis with 100 mmol L^–1^ lactate. (B) Identification and quantification of the lactate oxidation products (100 mmol L^–1^) after bulk electrolysis for 18 h at the Ru@Pt-CNT or Ru@Pt-CNT/OxOx electrode. Support electrolyte = 150 mmol L^–1^ phosphate-citrate buffer (pH = 5.2) and applied potential = 0.85 V vs Ag/AgCl. Peaks 1, 2, 3, 4, and 5 correspond to lactate, acetaldehyde, acetic acid, formic acid, and carbon dioxide.

Figure 2B summarizes the identification and concentration of the lactate oxidation products after 18 h of electrolysis for both electrodes. Thus, Figure 2B indicates that the hybrid system produced lactate electrooxidation products with a higher degree of oxidation (formic acid (*n*= 4 e^–^), and carbon dioxide (*n*= 12 e^–^)) compared to the system containing only metal catalysts (acetaldehyde (*n*= 2 e^–^), and acetic acid (*n* = 4 e^–^)).

Our findings indicate that the bicatalytic architecture of Ru@Pt-CNT/OxOx efficiently harvests all electrons from lactate, leading to enhanced LBFC performance through the consumption of substantial amounts of lactate. While Ru@Pt-CNT can oxidize multiple groups within the lactate molecule, OxOx previously required the formation of the acid (acetic acid) to function effectively. The enzyme action has been confirmed before for ethylene glycol (EG) oxidation in a hybrid bicatalytic configuration in the presence of Amino-TEMPO and OxOx.^17^ They demonstrated that OxOx cleaves the carbon–carbon bond of acid products (glycolic and glyoxylic acid).^17^ This provides subsidies to propose the action of the OxOx enzyme herein (Figure 2B). The pathway involves sequential oxidation through acetaldehyde, acetic acid, and formic acid, yielding CO_2_. Our findings revealed that these catalysts operate simultaneously, aligning with the results presented in this study.

Remarkably, despite the high degree of lactate electrooxidation observed for the Ru@Pt-CNT electrode, only the addition of OxOx allowed CO_2_ to be generated through complete lactate electrooxidation and collection of 12 electrons.

Consequently, the proposed electrode configuration emerges as a promising bioanode material characterized by high oxidation rates and selective conversion, making it well-suited for advancing enzymatic biofuel cell (BFC) technologies.

### 3.3 Power Density Tests

Figure 3 shows the power density tests performed for the Ru@Pt-CNT and Ru@Pt-CNT/OxOx electrodes in the absence or presence of lactate (Figure 3A). Without lactate, the residual power density was just 13 ± 2 μW cm^–2^. In the presence of lactate, the values shifted from 75 ± 4 μW cm^–2^ to 147 ± 6 μW cm^–2,^ respectively, for Ru@Pt-CNT and Ru@Pt-CNT/OxOx electrodes. No shift of the maximum current density was observed for either system.

**Figure 3:**
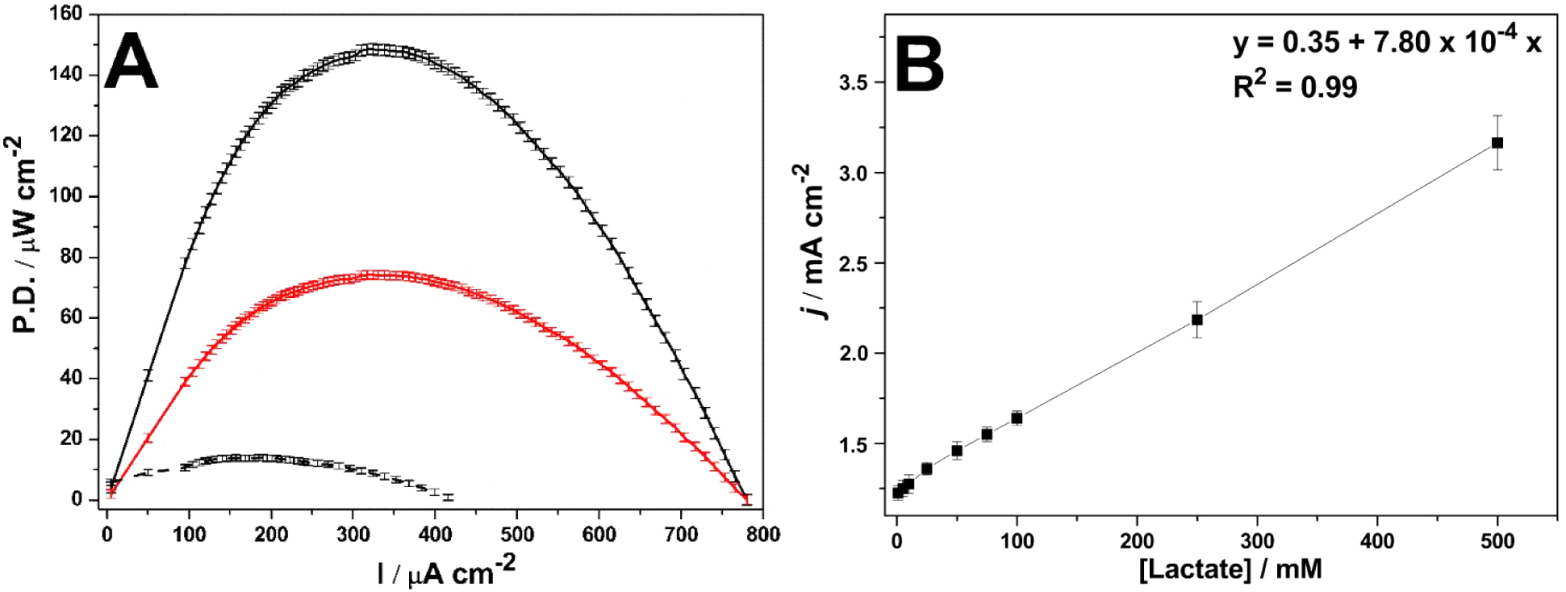
(A) LBFC power density curves for the Ru@Pt-CNT (solid red line) and Ru@Pt-CNT/OxOx (solid black line) electrodes in the presence of 100 mmol L^–1^ lactate. The test was also carried out for the hybrid electrode without lactate (dashed black line). (B) Maximum current density vs lactate concentration for the hybrid electrode. Supporting electrolyte of 150 mmol L^–1^ citric acid-phosphate buffers (pH = 5.2) and a scan rate of 1 mV s^–1^ at 25 °C.

The oxidation products identified in Figure 2, in conjunction with the electrochemical data presented in Figure 3A, demonstrate a significant enhancement in electron uptake and power density when the enzyme is incorporated into the bimetallic catalyst electrode. This enhancement is attributed to the hybrid system’s ability to complete lactate oxidation, facilitated by the cooperative activity between the enzyme and the metallic catalyst. The addition of the OxOx enzyme acted in the oxidation of acetic acid, a recalcitrant product for Pt-based electrocatalysts.^46^ Consequently, a 2-fold increase in power density is obtained when transitioning from a system capable of solely oxidizing the lactate oxygen group to a bicatalytic electrode capable of cleaving the carbon–carbon bonds in the intermediate oxidation products.

Figure 3B investigates the hybrid system’s performance by analyzing the maximum current density generated as the lactate concentration changes. The results show a linear increase in maximum current density with increasing lactate concentration. However, beyond 500 mmol L^–1^, the current density decreases due to saturation of the system by high lactate concentration, which impairs catalyst activity (Figure S2). The sensitivity of the hybrid system based on [lactate] vs current density presented a slope of 0.00078 mA·cm^–2^ L mol^–1^, demonstrating a good linear correlation (*R* ^2^ = 0.99). Furthermore, the low standard deviation confirms the satisfactory reproducibility of the approach. These findings highlight the potential application of the assembled electrode as a biosensor for lactate detection in the range of 1 to 500 mmol L^–1^, paving the way for developing improved and promising EBFC devices for real samples.

The stability of electrochemical cells operating with Ru@Pt-CNT and Ru@Pt-CNT/OxOx was assessed by conducting current density and power density tests under two conditions: (i) after 18 h of electrolysis and (ii) during a shelf life test. Table 1 summarizes the polarization data obtained from these stability tests.

**Table 1:** Stability Evaluation and Comparison of the Current Density and Power Density Generated for the Ru@Pt-CNT and Ru@Pt-CNT/OxOx Systems before and after Long-Term Electrolysis (18 h) and for 15, 30, 60, and 90 Days of Storage in a Refrigerator^a^

Anode Architecture	System Stability Test	*I*_max_ (μA cm^–2^)	Power Density (μW cm^–2^)
Ru@Pt-CNT	Freshly prepared	325 ± 9	75 ± 4
	After electrolysis	292 ± 9	68 ± 3
	After storage for 15 days	248 ± 10	57 ± 6
	After storage for 30 days	224 ± 8	51 ± 4
	After storage for 60 days	190 ± 5	44 ± 3
	After storage for 90 days	162 ± 6	37 ± 2
Ru@Pt-CNT/OxOx	Freshly prepared	330 ± 9	147 ± 6
	After electrolysis	314 ± 8	140 ± 7
	After storage for 15 days	283 ± 7	129 ± 5
	After storage for 30 days	255 ± 8	116 ± 7
	After storage for 60 days	230 ± 10	104 ± 5
	After storage for 90 days	205 ± 6	95 ± 3

a The concentration of lactate: 100 mmol L^–1^. Support electrolyte = 150 mmol L^–1^ phosphate-citrate buffer (pH = 5.2).

The Ru@Pt-CNT electrode experienced a 10% and 50% decrease in power density after electrolysis and 90 days of storage. In contrast, the cells operating with Ni@Pt-CNT/OxOx demonstrated exceptional stability, with a power density loss of only 5% following electrolysis. Moreover, the hybrid system exhibited remarkable long-term stability, as the power density decreased by only 35% after 90 days of storage.

These values demonstrate the enhanced stability of the hybrid system compared to the simple system when the enzyme is added to the electrode, which can be attributed to the hybrid system’s improved catalytic activity for lactate electrooxidation. Moreover, another crucial factor contributing to the electrodes’ stability is the incorporation of carbon nanotubes into the metallic catalyst structure.^15,47,48^

Remarkably, the hybrid electrode developed in this study demonstrated an unprecedented maximum power density for a lactate biofuel cell (BFC). The cooperative combination of a metallic catalyst and an enzyme capable of cleaving carbon–carbon bonds proved more efficient in lactate oxidation than previously reported systems employing enzymatic cascades^11,49^ or hybrid electrodes incorporating organic catalysts with lactate oxidase enzymes.^12^ Given the potential applications of this innovative BFC system, the hybrid bicatalytic electrode offers a promising platform for developing highly stable devices capable of efficient energy production and management through complete lactate oxidation.

### 3.4 Electrochemical Impedance Spectroscopy (EIS)

Figure 4 shows the EIS spectra of the Ru@Pt-CNT and Ru@Pt-CNT/OxOx electrodes in the presence of 100 mmol L^–1^ lactate at OCP (0.35 V vs Ag/AgCl). The Ru@Pt-CNT electrode achieved a 2-fold higher total impedance than the hybrid electrode (150 Ω vs 75 Ω, respectively). This indicated that the operational efficiency of the conversion process increased, given that the energy used by the system faced lower resistivity when OxOx was combined with the bimetallic composite on the electrode surface (Ohm’s first law). Besides that, a semicircle between 1000 and 100 Hz was achieved in the case of both electrodes. To evaluate the Ru@Pt-CNT electrode better, we used the equivalent circuit method to obtain the values associated with each element of the hybrid electrode. We simulated the two spectra by using the circuit represented in Figure 4D. The constant phase elements (CPEs) in parallel with *R*_CT_ were related to forming a double layer and the resistance associated with electron transfer, respectively. Here, we employed a CPE to emulate a nonideal capacitor on the electrode surface thanks to the double-layer loading. The CPEs reflect a nonideal homogeneous surface. That is, distortions might occur on the surface of both electrodes, resulting in the nonideality of the elements. In addition, other factors, such as current and charge leakage, could influence how the CPE behaves.^50^

**Figure 4:**
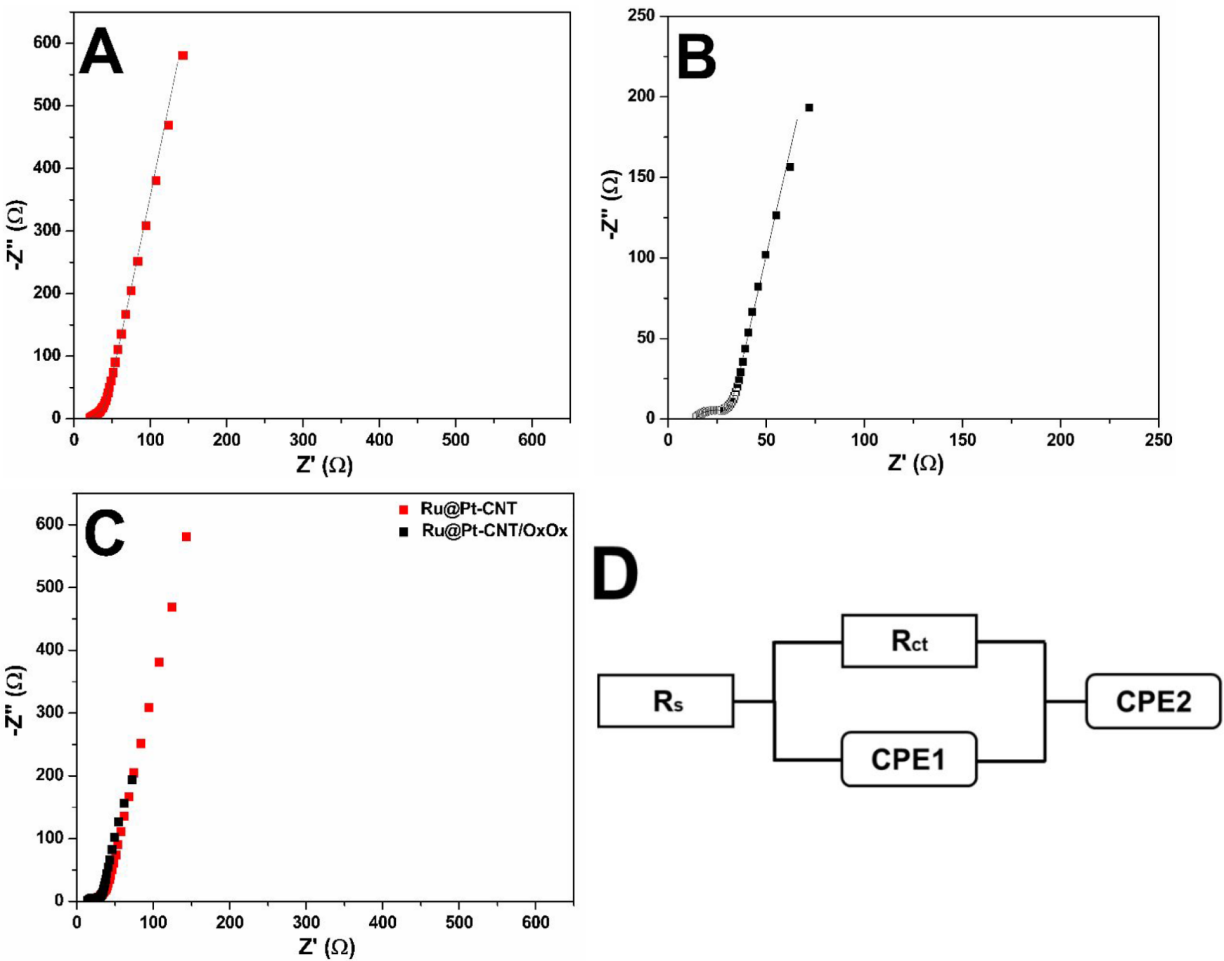
Impedance spectra performed at OCP (0.35 V vs Ag/AgCl) for the (A) Ru@Pt-CNT and (B) Ru@Pt-CNT/OxOx electrodes. (C) Comparison between the two electrodes. (D) An equivalent circuit was used to fit the measured impedance spectra. [lactate] = 100 mmol L^–1^ and support electrolyte = 150 mmol L^–1^ citric acid-phosphate buffer (pH = 5.2).

A simple Warburg element did not fit the experimental data, this might be explained due to the different convection processes and diffusion layer thicknesses.^51^ To fit the data in the presence of lactate a second CPE (CPE_2_) was added, in series with the CPE referring to the double layer (CPE_1_). CPE_2_ was purely associated with slow diffusion phenomena (the α values were close to 0.5), Table 2 shows the equivalent circuit values obtained for both electrodes operating at OCP.

**Table 2:** Equivalent Circuit Values for the Ru@Pt-CNT and Ru@Pt-CNT/OxOx Electrodes at OCP in the Presence of 100 mmol L^–1^ Lactate

**Element**	Ru@Pt-CNT	Ru@Pt-CNT/OxOx
*R*_s_	21.0 Ω	13.9 Ω
*R*_ct_	19.8 Ω	17.6 Ω
CPE 1		
α	0.894	0.884
*Y*_0_	9.20 mS·s^α^	3.51 mS·s^α^
CPE 2		
α	0. 592	0. 655
*Y*_0_	16.7 mS·s^α^	49.9 mS·s^α^

We also recorded EIS spectra for the Ru@Pt-CNT/OxOx electrode in the presence and absence of lactate at 0.85 V vs Ag/AgCl (Figure 5). In the absence of lactate, the hybrid electrode provides only a double-layer loading behavior associated with a diffusional process. However, in the presence of lactate, the spectrum of the hybrid electrode presented two defined semicircles, which corroborated with the CV and electrolysis experiments showing enhanced energy generation by combining the bimetallic composite and OxOx, allowing improved lactate bioelectrocatalytic oxidation.

**Figure 5:**
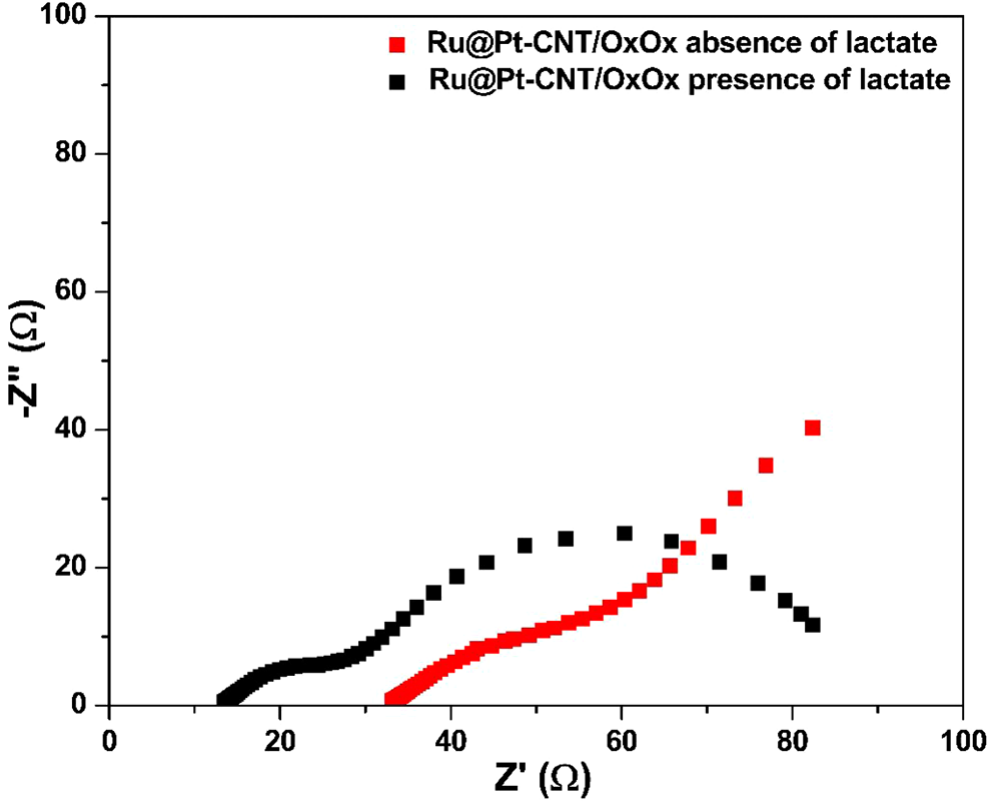
Nyquist plot for Ru@Pt-CNT/OxOx electrode in the presence (black line) and absence (red line) of 100 mmol L^–1^ lactate at 0.85 V (vs Ag/AgCl). Support electrolyte = 150 mmol L^–1^ citric acid-phosphate buffer (pH = 5.2).

Figure 6 shows a comparison of OxOx enzyme-containing and enzyme-free electrodes at operational potential 0.85 V (vs Ag/AgCl). The Bode plot (Figure 6A) was performed to improve the analysis of the total impedance generated by the two electrode configurations studied. A significant reduction in the total impedance of the catalyst anode was observed after the addition of the enzyme (225 Ω for the Ru@Pt-CNT electrode and 85 Ω for the hybrid system). The Bode plot confirmed that the presence of OxOx significantly reduces the total impedance of the electrode. It is also possible to observe that at frequencies from 1 to 100, there is an additional charge transfer to the system containing the OxOx enzyme, which may be associated with further reactions according to the proposed mechanism. The Nyquist plot (Figure 6B) confirms the influence of the enzyme on the hybrid system. The electrode without the enzyme (Ru@Pt-CNT) presented two poorly defined semicircles; one at high frequencies and the other one at lower frequencies. To fit the EIS experimental data a proposed circuit was used (Figure 6C). The first semicircle (CPE^1^ and *R*_CT_^1^), related to double-layer charging, is similar for both electrode configurations. The second semicircle, (CPE^2^ and *R*_CT_^2^) includes the action of a lower time constant occurring at both interfaces.^15^ The hybrid system achieved an *R*_CT_^2^ (48.5 Ω) which is 2-fold higher compared to the *R*_CT_^1^ (21.6 Ω). However, the presence of the hybrid film contributes to a decrease in the *R*_CT_^2^ 3.5 times compared with the metallic catalyst. The reduction of the total impedance of the bicatalytic hybrid electrode can be explained in the light of HPLC data, which reveals the formation of smaller and more polar molecules (formic acid, carbon dioxide).

**Figure 6:**
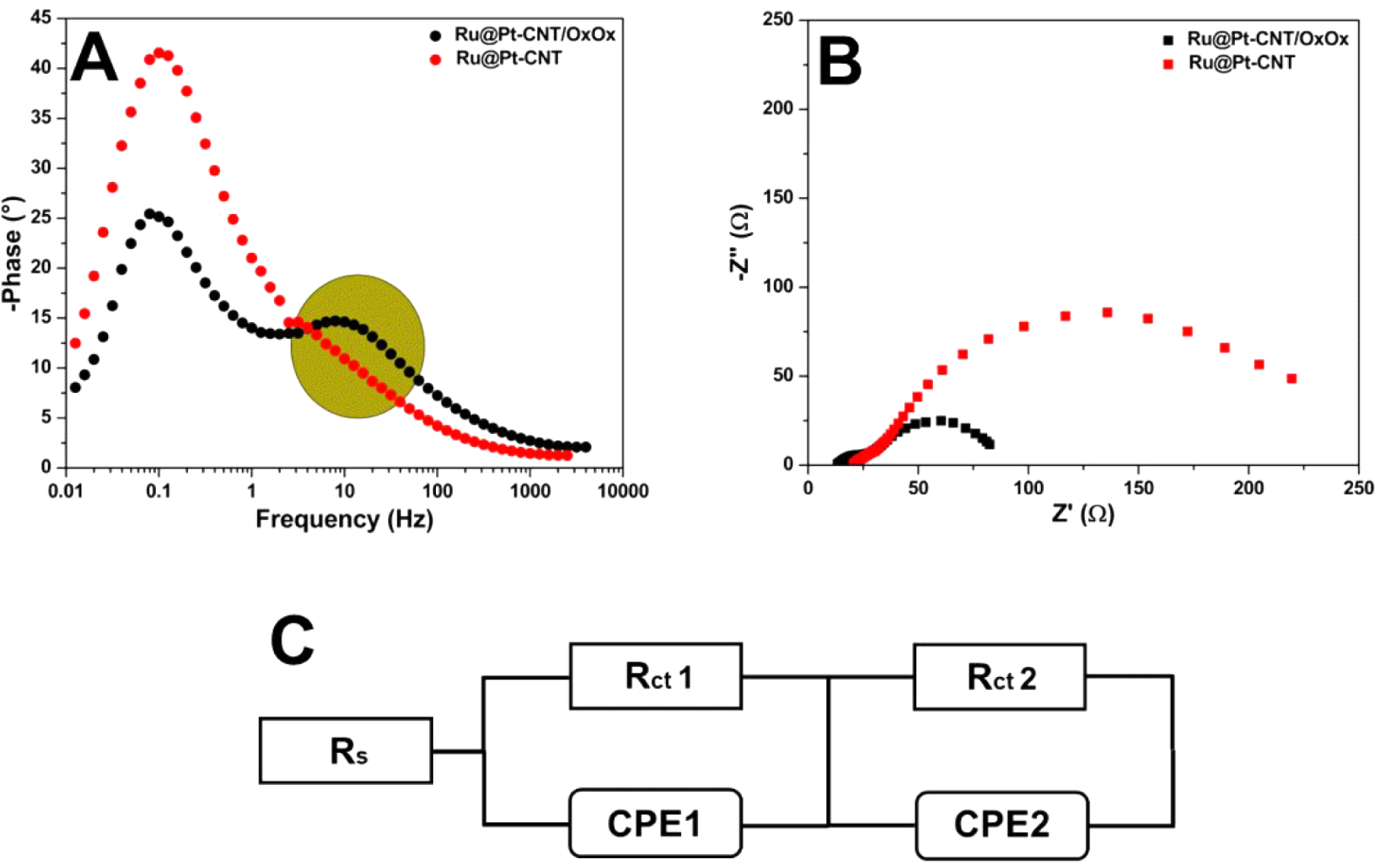
Bode (A) and Nyquist (B) plots depicting the difference in charge transfer between the Ru@Pt-CNT and Ru@Pt-CNT/OxOx electrodes at 0.85 V (vs Ag/AgCl). [lactate] = 100 mmol L^–1^ and support electrolyte = 150 mmol L^–1^ citric acid-phosphate buffer (pH = 5.2). (C) Equivalent circuit used to fit the measured impedance spectra.

Table 3 depicts the values attributed to each element of the circuit using the equivalent circuit and spectrum data obtained in Figure 6C.

**Table 3:** Values of the Equivalent Circuit Elements for the Ru@Pt-CNT and Ru@Pt-CNT/OxOx System Obtained in Operational Potential (0.85 V *vs* in the Presence of Lactate

Ru@Pt-CNT			Ru@Pt-CNT/OxOx		
**Element**		**Value**	**Element**		**Value**
*R*_s_		20.3 Ω	*R*_s_		13.2 Ω
*R*_CT_^1^		30.2 Ω	*R*_CT_^1^		21.6 Ω
CPE^**1**^	α^1^	0.533	CPE^1^	α^1^	0.593
	*Y*_0_^1^	13.4 mS·s^α^		*Y*_0_	7.58 mS·s^α^
*R*_CT_^2^		169 Ω	*R*_CT_^2^		48.5 Ω
CPE^**2**^	α^2^	0.998	CPE^**2**^	α^2^	0.998
	*Y*_0_^2^	22.8 mS·s^α^		*Y*_0_^2^	60.6 mS·s^α^

## 4 Conclusion

We achieved complete lactate electrooxidation by using a hybrid electrode containing a bimetallic composite (Ru@Pt-CNT) and the enzyme oxalate oxidase (OxOx) on a carbon paper surface. The hybrid electrode furnished high catalytic activity (1.5 ± 0.1 mA cm^–2^) and power density (147 ± 6 μW cm^–2^). The electrochemical results confirmed that the bimetallic composite efficiently produced energy by replacing many enzymes involved in multistep cascade reactions. Furthermore, the cooperative action between Ru@Pt-CNT and OxOx improved electrode stability and catalytic performance during long-term electrolysis compared to the electrode containing only Ru@Pt-CNT. The hybrid electrode collected 12 electrons from lactate and completely oxidized it to CO_2_. EIS results pave the way for obtaining necessary information about the action of OxOx and the Ru@Pt-CNT nanoparticles through lactate electrooxidation. Hybrid LBFC preparation was inexpensive, fast, and simple and provided a system with outstanding electrochemical performance, which could facilitate the development of efficient and highly sensitive biosensors. Overall, the Ru@Pt-CNT/OxOx electrode represents a promising advancement of new LBFCs, allowing the implementation of energy harvesting devices.

## Supplementary Material



## Notes

Comparison of the maximum power density and maximum current for different hybrid EBFCs (Table S1); comparison of lactate consumption before and after 18 h bulk electrolysis using Ru@Pt-CNT and Ru@Pt-CNT/OxOx electrodes (Figure S1); maximum current density vs lactate concentration (Figure S2); references cited in Table 1 (9 references) (PDF)
