# Supplementary material for: Enhanced Biofuel Cells Based on a Hybrid Enzymatic/Bimetallic Composite for Complete Lactate Catalytic Electrooxidation
Source: ACS Mater Au. 2025 Jun 9;5(4):732–42. doi: 10.1021/acsmaterialsau.5c00039 (PMC12257398; doi:10.1021/acsmaterialsau.5c00039)
Supplement: Supplementary file 1 [file mg5c00039_si_001.pdf]

Enhanced Biofuel Cell based on a Hybrid Enzymatic/Bimetallic Composite for Complete Lactate Catalytic Electrooxidation

Jefferson Honorio Franco[a,b], João Victor Bonaldo[a], Shelley D. Minteer[c,d], Adalgisa R. De Andrade[a,b*]

[a] Department of Chemistry, Faculty of Philosophy Sciences and Letters at Ribeirão Preto, University of São Paulo, 14040-901 Ribeirão Preto, SP, Brazil;

[b] UNESP, National Institute for Alternative Technologies of Detection, Toxicological Evaluation and Removal of Micropollutants and Radioactives (INCT-DATREM), Institute of Chemistry, P.O. Box 355, 14800-900, Araraquara, SP, Brazil;

[c] Department of Chemistry, University of Utah, Salt Lake City, UT 84112, United States.

[d] Department of Chemistry, Missouri University of Science and Technology, Rolla, MO 65401, United States.

* Author to whom correspondence should be addressed: E-Mail: ardandra@usp.br

Table S1. Comparison of the maximum power density and maximum current for different hybrid EBFCs.

Electrode	Fuel	Power Density (μA/cm^{-2})	J_{max} (mA/cm^{-2})	Reference
**Ru@Pt-CNT/OxOx**	Lactate	147.0	1.50	**This work**
**TEMPO-NH_2/OxDc**	Lactate	*	0.15	[1]
**Ni@Pt-CNT/OxOx**	Glucose	400.0	1.30	[2]
**MWCNT-COOH/Pyrene-TEMPO/OxOx**	Ethylene glycol	285.0	0.17	[3]
**$Pt_{65}Sn_{35}$/MWCNTs+OxOx**	Ethylene glycol	332.0	2.45	[4]
**MWCNT-COOH/TEMPO-LPEI/OxOx**	Ethanol	302.5	2.35	[5]
**MWCNT-COOH/Pyrene-TEMPO/OxDc**	Ethanol	388.0	2.80	[6]
**TEMPO-NH_2/OxDc**	Ethanol	78.0	0.56	[7]
**TEMPO-NH_2/OxOx**	Glycerol	*	0.88	[8]
**MWCNT/TEMPO-LPEI/OxDc**	Glycerol	*	1.30	[9]

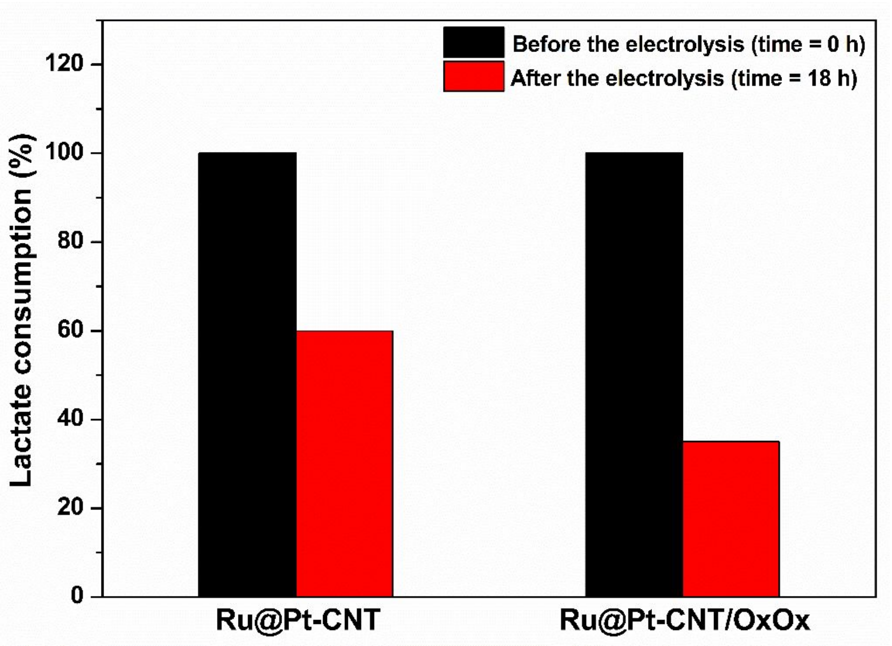

Figure S1. Comparison of lactate consumption before and after 18 hours bulk electrolysis using Ru@Pt-CNT and Ru@Pt-CNT/OxOx electrodes.

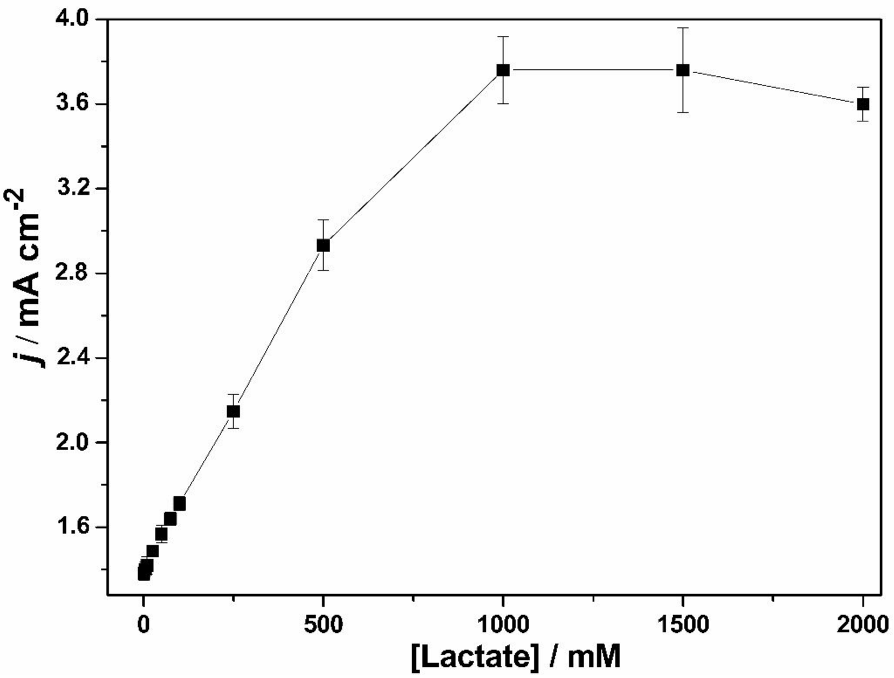

Figure S2. Maximum current density *vs* lactate concentration (1 – 2000 mM) for the hybrid electrode Ru@Pt-CNT/OxOx. Supporting electrolyte of 150 mmol L^{-1} citric acid-phosphate buffers (pH = 5.2) and a scan rate of 1 mV s^{-1} at 25 °C.

References

(1) Franco, J. H.; Grattieri, M.; de Andrade, A. R.; Minteer, S. D. Unveiling Complete Lactate Oxidation through a Hybrid Catalytic Cascade. *Electrochim. Acta* **2021**, *376*, 138044, DOI: 10.1016/j.electacta.2021.138044.

(2) Franco, J. H.; Bonaldo, J. V.; da Silva, R. G.; Minteer, S. D.; De Andrade, A. R. Development of an Enhanced Hybrid Bi-Catalytic Electrode Containing Bimetallic Composite Catalyst and Immobilized Enzyme for Complete Glucose Electrooxidation. *Biosens. Bioelectron.: X* **2023**, *14*, 100354, DOI: 10.1016/j.biosx.2023.100354.

(3) Franco, J. H.; Bonaldo, J. V.; Minteer, S. D.; De Andrade, A. R. Assembly of an Improved Hybrid Cascade System for Complete Ethylene Glycol Oxidation: Enhanced Catalytic Performance for an Enzymatic Biofuel Cell. *Biosens. Bioelectron.* **2022**, *216*, 114649, DOI: 10.1016/j.bios.2022.114649.

(4) Antonio, J. G. R.; Franco, J. H.; Almeida, P. Z.; Almeida, T. S.; Teixeira de Morais Polizeli, M. de L.; Minteer, S. D.; Rodrigues de Andrade, A. Carbon Nanotube PtSn Nanoparticles for Enhanced Complete Biocatalytic Oxidation of Ethylene Glycol in Biofuel Cells. *ACS Materials Au* **2022**, *2* (2), 94–102, DOI: 10.1021/acsmaterialsau.1c00029.

(5) Franco, J. H.; de Almeida, P. Z.; Abdellaoui, S.; Hickey, D. P.; Ciancaglini, P.; de Lourdes T. M. Polizeli, M.; Minteer, S. D.; de Andrade, A. R. Bioinspired Architecture of a Hybrid Bifunctional Enzymatic/Organic Electrocatalyst for Complete Ethanol Oxidation. *Bioelectrochemistry* **2019**, *130*, 107331, DOI: 10.1016/j.bioelechem.2019.107331.

(6) Franco, J. H.; Klunder, K. J.; Lee, J.; Russell, V.; de Andrade, A. R.; Minteer, S. D. Enhanced Electrochemical Oxidation of Ethanol Using a Hybrid Catalyst Cascade Architecture Containing Pyrene-TEMPO, Oxalate Decarboxylase and Carboxylated Multi-Walled Carbon Nanotube. *Biosens. Bioelectron.* **2020**, *154*, 112077, DOI: 10.1016/j.bios.2020.112077.

(7) Franco, J. H.; Klunder, K. J.; Russell, V.; de Andrade, A. R.; Minteer, S. D. Hybrid Enzymatic and Organic Catalyst Cascade for Enhanced Complete Oxidation of Ethanol in an Electrochemical Micro-Reactor Device. *Electrochim. Acta* **2019**, *331*, 135254, DOI: 10.1016/j.electacta.2019.135254.

(8) Hickey, D. P.; McCammant, M. S.; Giroud, F.; Sigman, M. S.; Minteer, S. D. Hybrid Enzymatic and Organic Electrocatalytic Cascade for the Complete Oxidation of Glycerol. *J. Am. Chem. Soc.* **2014**, *136* (45), 15917–15920, DOI: 10.1021/ja5098379.

(9) Macazo, F. C.; Hickey, D. P.; Abdellaoui, S.; Sigman, M. S.; Minteer, S. D. Polymer-Immobilized, Hybrid Multi-Catalyst Architecture for Enhanced Electrochemical Oxidation of Glycerol. *Chem. Comm.* **2017**, *53* (74), 10310–10313, DOI: 10.1039/C7CC05724E.
